# Retinal vessel caliber and cognitive performance: the multi-ethnic study of atherosclerosis (MESA)

**DOI:** 10.1038/s41598-024-54412-2

**Published:** 2024-02-19

**Authors:** Nada El Husseini, Christopher L. Schaich, Suzanne Craft, Stephen R. Rapp, Kathleen M. Hayden, Richey Sharrett, Mary Frances Cotch, Tien Y. Wong, Jose A. Luchsinger, Mark A. Espeland, Laura D. Baker, Alain G. Bertoni, Timothy M. Hughes

**Affiliations:** 1 Department of Neurology, Duke University Medical Center, Duke South, Purple Zone, Suite 0109, Durham, NC 27710 USA; 2 Department of Surgery, Hypertension and Vascular Research Center, Wake Forest University School of Medicine, Winston Salem, NC USA; 3 Gerontology and Geriatric Medicine, Wake Forest University School of Medicine, Winston Salem, NC USA; 4 Psychiatry and Behavioral Medicine, Wake Forest University School of Medicine, Winston Salem, NC USA; 5 Social Sciences and Health Policy, Wake Forest University School of Medicine, Winston Salem, NC USA; 6 Department of Epidemiology, Johns Hopkins University, Baltimore, MD USA; 7 National Eye Institute (NIH), Bethesda, MD USA; 8 Department of Ophthalmology and Visual Sciences, National University of Singapore, Singapore, Singapore; 9 Tsinghua Medicine, Tsinghua University, Beijing, China; 10 Division of General Medicine, Columbia University Medical Center, New York, NY USA; 11 Epidemiology and Prevention, Wake Forest University School of Medicine, Winston Salem, NC USA

**Keywords:** Cognitive neuroscience, Retina

## Abstract

Retinal vessel calibers share anatomic and physiologic characteristics with the cerebral vasculature and can be visualized noninvasively. In light of the known microvascular contributions to brain health and cognitive function, we aimed to determine if, in a community based-study, retinal vessel calibers and change in caliber over 8 years are associated with cognitive function or trajectory. Participants in the Multi-Ethnic Study of Atherosclerosis (MESA) cohort who completed cognitive testing at Exam 5 (2010–2012) and had retinal vascular caliber measurements (Central Retinal Artery and Vein Equivalents; CRAE and CRVE) at Exam 2 (2002–2004) and Exam 5 were included. Using multivariable linear regression, we evaluated the association of CRAE and CRVE from Exam 2 and Exam 5 and their change between the two exams with scores on tests of global cognitive function (Cognitive Abilities Screening Instrument; CASI), processing speed (Digit Symbol Coding; DSC) and working memory (Digit Span; DS) at Exam 5 and with subsequent change in cognitive scores between Exam 5 and Exam 6 (2016–2018).The main effects are reported as the difference in cognitive test score per SD increment in retinal vascular caliber with 95% confidence intervals (CI). A total of 4334 participants (aged 61.6 ± 9.2 years; 53% female; 41% White) completed cognitive testing and at least one retinal assessment. On multivariable analysis, a 1 SD larger CRAE at exam 5 was associated with a lower concomitant CASI score (− 0.24, 95% CI − 0.46, − 0.02). A 1 SD larger CRVE at exam 2 was associated with a lower subsequent CASI score (− 0.23, 95%CI − 0.45, − 0.01). A 1 SD larger CRVE at exam 2 or 5 was associated with a lower DSC score [(− 0.56, 95% CI − 1.02, − 0.09) and − 0.55 (95% CI − 1.03, − 0.07) respectively]. The magnitude of the associations was relatively small (2.8–3.1% of SD). No significant associations were found between retinal vessel calibers at Exam 2 and 5 with the subsequent score trajectory of cognitive tests performance over an average of 6 years. Wider retinal venular caliber was associated with concomitant and future measures of slower processing speed but not with later cognitive trajectory. Future studies should evaluate the utility of these measures in risk stratification models from a clinical perspective as well as for screening on a population level.

## Introduction

Cerebrovascular disease is a risk factor for cognitive impairment and dementia in older adults^1,2^. Through common underlying pathophysiological processes, extracranial vascular markers of microvascular disease may convey information about brain health^3^. Microvascular findings in the retina are of particular interest because they can be directly visualized noninvasively and share anatomic and physiologic characteristics with cerebral vasculature^3^. For example, retinal microvascular abnormalities such as focal narrowing, arteriovenous nicking, arteriolar narrowing and venular dilatation are variably associated with stroke, white matter lesions, cerebral microbleeds, and brain atrophy^3–7^. It is unclear to what degree retinal vascular calibers are gross manifestations of anatomical parameters that are present at birth or develop over time when exposed to other biological or external insults.

Several cross-sectional studies have described associations between retinal microvascular signs and cognitive impairment^3^. For example, reduced retinal vascular fractal dimensions have been associated with poorer performance in global cognitive function and in the specific domains of verbal memory, visuoconstruction and visuomotor speed as well as with Alzheimer’s dementia^8,9^. However, it remains unclear whether certain retinal microvascular findings can be used as an early marker for future cognitive impairment or whether certain characteristics of the retinal vasculature may warrant screening for brain health. In the Atherosclerosis Risk in Communities (ARIC) longitudinal study of baseline retinal microvascular abnormalities and follow-up cognitive assessment 14 years later, retinopathy rather than retinal vascular caliber was associated with decline in executive function and psychomotor speed, but retinal vessels were only evaluated at baseline so no conclusions could be made about the association of changes in vessel caliber with cognitive outcome^10^.

There is ongoing interest to discover simple and cost-effective biomarkers to be deployed in multiple settings that would help with detection of early dementia or stratify individuals at high risk for dementia^2^. The Multi-Ethnic Study of Atherosclerosis (MESA) cohort offers a unique opportunity to evaluate the cross-sectional and longitudinal association of baseline and follow-up retinal microvascular caliber measurements, collected up to 8 years apart, with global cognitive function, processing speed and working memory as well as with subsequent change in cognitive function in an ethnically and racially diverse community-based cohort.

## Methods

### Participants

MESA is a prospective observational cohort of 6814 participants aged 45–84 years at the baseline examination (2000–2002) who self-reported their race/ethnicity as non-Hispanic White, non-Hispanic Black, Hispanic, or Chinese^11^. Participants free of clinically apparent cardiovascular disease (CVD) were recruited from six US communities: Baltimore City and Baltimore County, Maryland; Chicago, Illinois; Forsyth County, North Carolina; Los Angeles County, California; Northern Manhattan and the Bronx, New York; and St. Paul, Minnesota^11^. The present study focuses on participants who completed cognitive testing at Exam 5 (2010–2012) and Exam 6 (2016–2018) and had retinal vascular caliber measurements at Exam 2 (2002–2004) and/or Exam 5. The MESA protocol was approved by the Institutional Review Boards of all participating institutions and by the National Heart, Lung, and Blood Institute and conducted according to relevant guidelines and regulations. All participants in MESA signed informed consent.

### Measurements

At each MESA examination, data were collected using standardized questionnaires to assess self-reported demographics (age, sex, race/ethnicity, level of education, smoking status) and medication usage for high blood pressure, high cholesterol, or diabetes. Participants were interviewed and tested in the language of their choice, including English, Spanish, or Chinese (Mandarin or Cantonese).

Clinical data from Exam 2 were used. Resting brachial systolic blood pressure (SBP) and diastolic blood pressure (DBP) measurements were obtained using the Dinamap® automated blood pressure device (Dinamap Monitor Pro 100®); three sequential measures were obtained, and the average of the second and third measurements was recorded. Hypertension was defined as a systolic blood pressure ≥ 140 mmHg or diastolic blood pressure ≥ 90 mmHg or on the basis of the medication inventory including blood pressure medicine and a self-report of hypertension. Total and high-density lipoprotein (HDL) cholesterol, triglycerides and glucose were measured from blood samples obtained after a 12-h fast. Low-density lipoprotein (LDL) cholesterol was calculated with the Friedewald equation among those with triglycerides less than 400 mg/dL. Diabetes was defined as fasting glucose ≥ 7 mmol/L (126 mg/dL) or use of hypoglycemic medication. Body Mass Index (BMI) on Exam 2 was calculated as weight (kg) divided by the square of height (m^2^). The ascertainment of incident stroke events between Exams 2 and 5 was based on telephone follow-up calls and hospital records, with stroke determined by adjudication.

Alcohol consumption and cigarette smoking were assessed at Exam 2 by participant’s response to a personal history questionnaire. Alcohol use was screened with the following questions: “Have you ever consumed alcoholic beverages?” and “Do you presently drink alcoholic beverages?” Similar questions were used to assess smoking behavior and participants were classified as never, former, or current smokers. Smoking intensity was captured by the number of pack-years.

C-reactive protein (CRP) was measured using the BNII nephelometer (N High-Sensitivity CRP); intra-assay coefficient of variation for CRP range from 2.3 to 4.4% and inter-assay coefficients of variation range from 2.1 to 5.7%^12^. For the urine albumin and creatinine ratio measurement, a spot urine sample was collected. Urine albumin and creatinine were measured using nephelometry and the Jaffe method, respectively. Urine albumin-creatinine ratio (ACR) was calculated and was categorized as follows: (1) no albuminuria (urine ACR < 17 mg/g for men and < 25 mg/g for women); (2) microalbuminuria (urine ACR between 17 to 249 mg/g for men and 25 to 349 mg/g for women); and (3) macroalbuminuria (urine ACR ≥ 250 mg/g for men and ≥ 355 mg/g for women)^13^. Albuminuria for this analysis was defined as presence of either microalbuminuria or macroalbuminuria.

### Retinal measurements

Retinal photography was performed using a standardized protocol^14,15^. Photographic fields of optic disc and macula of both eyes of each participant were photographed through non-pharmacologically dilated pupils using a 45-degree 6.3-megapixel digital nonmydriatic camera. These photographs were sent from all six centers to a central site at the University of Wisconsin-Madison for measurement of retinal vascular caliber and evaluation of other retinal pathology. Trained graders at these centralized sites were blinded to participant characteristics^16,17^. For each image, all arterioles and venules coursing through an area one-half to one-disc diameter from the optic disc margin were measured using a computer-based program (IVAN, University of Wisconsin, Madison), based on a detailed protocol^16–18^. Retinal arteriolar caliber was summarized as the central retinal artery equivalent (CRAE), while retinal venular caliber was summarized as central retinal vein equivalent (CRVE)^16,19^. The CRAE and CRVE equivalents are the projected caliber for the central retinal artery/vein, measured away from the optic disc.

In MESA, the CRAE and CRVE values from the right eye were used. When the right eye values could not be assessed due to missing images or poor image quality, the left eye values were used instead. High correlation of retinal vessel diameters between eyes has been previously established^17^. Retinal vessel calibers and their change from Exam 2 to Exam 5 were normally distributed (Supplemental Figs. 1–4).

### Assessment of cognition

Cognitive function was evaluated at Exam 5 (2010–2012) and Exam 6 (2016–2018) using three standardized and validated tests including the following: Cognitive Abilities Screening Instrument (CASI, version 2), a measure of global cognitive functioning; Digit Symbol Coding, a test of processing speed; and Digit Span (forward and backward combined), a test of working memory, each previously described in detail^20^.

Briefly, the CASI (scored 0–100; lower score indicates worse performance) includes 25 items representing 9 cognitive domains: attention, concentration, orientation, language, verbal fluency, visual construction, abstraction/judgment, and short- and long-term memory. The Digit Symbol Coding (scored 0–133) and Digit Span (scored 0–28) are subtests of the Wechsler Adult Intelligence Scale-III, with lower scores representing poorer performance^21^. For the Digit Symbol Coding, participants were presented 9 digit-symbol pairs followed by a list of randomly ordered digits, below which they were asked to write as many corresponding symbols as possible within 120 s. For the Digit Span, participants were asked to repeat increasing spans of random numbers both forward and backward, for a maximum of 14 trials in each direction. Forward and backward scores were summed to provide a total score ranging from 0 to 28^22,23^. For all tests, a higher score represents better cognitive function. Cognitive data were excluded from the current analysis if marked invalid by the test administrator at the time of testing or in the setting of incomplete data resulting in CASI score < 20.

### Statistical analysis

**Figure 1:**
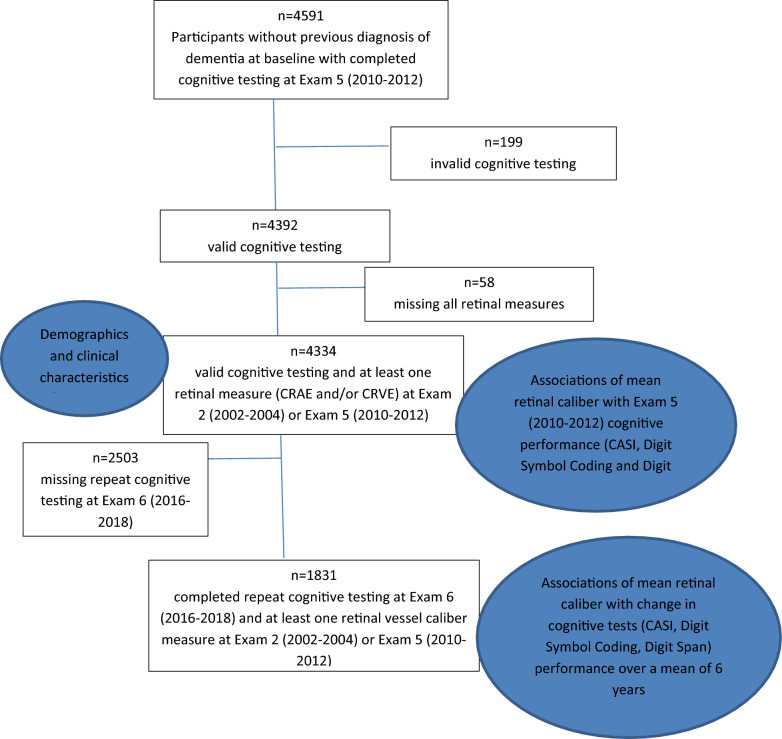
Study Participants and associated analyses.

MESA participants without clinically diagnosed dementia at baseline (by patient report of a diagnosis by a physician) and with both valid cognitive testing at Exam 5 and at least CRAE or CRVE from Exam 2 and/or Exam 5 were included in the analytic sample (Fig. 1). First, we compared the demographic and clinical characteristics of included and excluded participants using t-tests for continuous and Pearson chi-square tests for categorical variables. We then used multivariable linear regression with inverse probability weighting for exclusion to assess the association of retinal caliber measures at Exam 2, Exam 5, and their change with Exam 5 CASI, Digit Symbol Coding, and Digit Span test scores. Based on differences in the included and excluded participants (Table 1), we used inverse probability weighting in our regression models to mitigate the potential bias from differential exclusion. We assessed unadjusted associations, demographics-adjusted linear models (age, sex, race/ethnicity, and completion of high school or more; Model 1), and models additionally adjusted for cardiovascular and cognitive risk factors (body mass index [BMI], hypertension, diabetes, current smoking status, current alcohol consumption status, optimal [< 100 mg/dL] vs. not-optimal [≥ 100 mg/dL] low-density lipoprotein levels, c-reactive protein (CRP), albuminuria, and incident stroke between Exam 2 and Exam 5; Model 2). Regression diagnostics were assessed visually via residual, normal Q-Q, and scale-location plots to ensure assumptions for linearity, normal distribution of residuals, and homoscedasticity were met.

**Table 1:** Demographic and clinical characteristics of MESA participants included in the study compared to participants excluded based on missing data.

	Included	Excluded	*p*
N	4334	257	
Age at Exam 2, years, mean (SD)	61.6 (9.2)	67.4 (10.7)	< 0.001
Female, %	2309 (53.3)	131 (51.0)	0.472
Race, %			< 0.001
White	1795 (41.4)	73 (28.4)	
African–American	1140 (26.3)	71 (27.6)	
Hispanic	922 (21.3)	62 (24.1)	
Chinese–American	477 (11.0)	51 (19.8)	
Education, high school or higher, %	3753 (86.7)	181 (70.7)	< 0.001
Body mass index, kg/m^2^, mean (SD)	28.5 (5.5)	28.0 (5.9)	0.258
Hypertension, %	1776 (41.4)	136 (53.1)	0.002
Diabetes, %	549 (12.7)	48 (19.0)	0.004
Alcohol, % current use	2338 (55.0)	104 (43.2)	< 0.001
Smoking, % current	460 (10.9)	19 (7.9)	0.139
Hemoglobin A1c, %, mean (SD)	5.7 (0.9)	5.9 (1.0)	< 0.001
LDL cholesterol, % optimal (< 100 mg/dl)	1435 (33.6)	104 (42.3)	0.005
Urine albuminuria, %	334 (7.9)	28 (11.8)	0.033
C-reactive protein, mg/L, mean (SD)	3.6 (5.1)	3.3 (4.8)	0.440
Incident stroke between Exam 2 and 5, n (%)	77 (1.8)	8 (3.1)	0.123
Exam 2 CRAE, µm, mean (SD)	144.4 (14.0)	142.7 (13.8)	0.104
Exam 5 CRAE, µm, mean (SD)	141.5 (14.1)	140.2 (14.4)	0.258
Change CRAE, µm, mean (SD)	− 2.96 (11.4)	− 2.87 (10.8)	0.928
Exam 2 CRVE, µm, mean (SD)	214.0 (21.6)	214.1 (22.1)	0.955
Exam 5 CRVE, µm, mean (SD)	206.4 (21.5)	207.1 (23.1)	0.677
Change CRVE, µm, mean (SD)	− 7.57 (14.8)	− 7.32 (15.4)	0.842
Cognitive Abilities Screening Instrument (CASI), mean (SD)	88.0 (8.3)	67.8 (27.7)	< 0.001
Digit Symbol Coding, mean (SD)	50.9 (18.2)	37.3 (20.9)	< 0.001
Digit Span, mean (SD)	15.3 (4.5)	13.4 (4.3)	< 0.001

Retinal calibers were standardized (mean = 0 and SD = 1) in all analyses. Exam 2 retinal calibers were adjusted for covariates measured at Exam 2, and Exam 5 retinal calibers and change in retinal calibers were adjusted for covariates measured at Exam 5. Changes in retinal calibers were additionally adjusted for their baseline (Exam 2) values, including in otherwise unadjusted models. CRAE and CRVE were not included in the same model because of collinearity (Pearson r = 0.60, *p* < 0.0001). Stabilized inverse probability weights were generated via logistic regression based on inclusion in the analysis for each cognitive test, conditioned on Model 2 covariates at Exam 2 or Exam 5 depending on the retinal caliber measure of interest in each model. Thus, weights were specific to each cognitive test outcome and retinal caliber time point (Exam 2 or Exam 5). Missing covariate data, but not CRVE, CRAE or cognitive test scores, were imputed using multivariate imputation by chained equations. Continuous and categorical covariates were both imputed via the classification and regression trees (CART) process. Results from 10 iterations of 20 imputed datasets were combined for validity. Missing data did not exceed 2.7% for any covariate. We then used these multivariable methods to evaluate the association of each retinal caliber measure with the change in each cognitive test score from Exam 5 to Exam 6, a mean (SD) period of 6.3 (0.5) years. These analyses were adjusted for Model 2 covariates and the respective Exam 5 test score. Separate sets of stabilized inverse probability weights were generated for providing repeat CASI, Digit Symbol Coding, or Digit Span test data at Exam 6. A comparison of standardized mean differences between weighted and unweighted continuous variables indicates that better balance was achieved by inverse probability weighting in our multivariable models (Supplemental Tables 1, 2). We also assessed effect modification by sex and race/ethnicity by including interaction terms in models.

We report main effects as the difference in cognitive test score per SD increment in retinal vascular caliber with 95% confidence intervals (CI). Standardized effect sizes for the significant associations, which are the regression coefficient as a percent of the dependent variable SD, were calculated. Analyses were completed in R 4.1.2 (R Foundation, 2021), and multiple imputation was performed using the mice package (version 3.15) for R.

### Ethics approval and consent to participate

The MESA protocol was approved by the Institutional Review Boards of all participating institutions and by the National Heart, Lung, and Blood Institute according to relevant guidelines and regulations. All participants in MESA signed informed consent.

## Results

A total of 4334 participants had valid cognitive testing, and at least one retinal caliber measurement from Exam 2 or Exam 5 (Fig. 1). Differences in the baseline characteristics between participants included in this study and those excluded based on incomplete or invalid data are shown in Table 1.

Excluded participants tended to have more hypertension, diabetes and albuminuria, and lower cognitive scores (Table 1). At the time of retinal imaging and cognitive testing at Exam 5, the mean (SD) age of participants was 61.6 (9.2) years, 53% were women, and 41% were non-Hispanic White. Incident stroke occurred in 77 participants between Exams 2 and 5. Other demographics and clinical characteristics from Exam 2 are described in Table 1. The mean (SD) change in CRAE over an average of 8 years was − 2.96 µm (11.4) and the mean (SD) change in CRVE was − 7.57 µm (14.8).

### Global cognitive performance

Associations between measures of retinal caliber and cognitive tests are shown in Table 2. In fully adjusted models, 1-SD increments in CRAE at Exam 5 and CRVE at Exam 2 were associated with lower global cognitive performance as measured by the CASI (− 0.24 [95% CI − 0.46, − 0.02] and − 0.23 [95% CI − 0.45, − 0.01] points, respectively). Additionally, an increase in CRAE from Exam 2 to Exam 5 was associated with worse global cognitive performance at Exam 5 (− 0.25 [95% CI − 0.48, − 0.02] points; Table 2). However, a 1-SD increase in CRVE from Exam 2 to Exam 5 was prospectively associated with a 0.33 (95% CI 0.01, 0.66) score increase in the CASI at Exam 6 (Table 3). No other retinal caliber measures were associated with changes in CASI performance.

**Table 2:** Associations of retinal caliber with Exam 5 cognitive performance (CASI, Digit Symbol Coding and Digit Span) among participants with at least one retinal caliber measurement.

	Difference in Exam 5 test score per SD increment in retinal caliber (95% CI)			
	n	Unadjusted	Model 1	Model 2
CASI^a^				
CRAE				
Exam 2	4155	0.01 (− 0.24, 0.27)	0.003 (− 0.21, 0.22)	0.02 (− 0.20, 0.23)
Exam 5	3977	− **0.45 (**− **0.70, **− **0.19)**	− **0.28 (**− **0.49, **− **0.06)**	− **0.24 (**− **0.46, **− **0.02)**
Change	3816	− **0.60 (**− **0.88, **− **0.32)**	− **0.29 (**− **0.53, **− **0.06)**	− **0.25 (**− **0.48, **− **0.02)**
CRVE				
Exam 2	4171	− **0.85 (**− **1.10, **− **0.60)**	− **0.29 (**− **0.51, **− **0.07)**	− **0.23 (**− **0.45, **− **0.01)**
Exam 5	4006	− **0.56 (**− **0.81, **− **0.31)**	− **0.26 (**− **0.48, **− **0.03)**	− 0.21 (− 0.44, 0.01)
Change	3852	0.10 (− 0.17, 0.38)	− 0.03 (− 0.25, 0.20)	− 0.03 (− 0.26, 0.20)
Digit symbol coding^b^				
CRAE				
Exam 2	3797	0.51 (− 0.07, 1.09)	− 0.10 (− 0.55, 0.36)	− 0.06 (− 0.52, 0.40)
Exam 5	3642	0.16 (− 0.43, 0.76)	− 0.06 (− 0.53, 0.41)	0.07 (− 0.40, 0.54)
Change	3499	− 0.41 (− 1.07, 0.24)	− 0.16 (− 0.67, 0.35)	0.06 (− 0.44, 0.56)
CRVE				
Exam 2	3814	− **1.59 (**− **2.15, **− **1.03)**	− **0.89 (**− **1.34, **− **0.43)**	− **0.56 (**− **1.02, **− **0.09)**
Exam 5	3672	− **0.96 (**− **1.55, **− **0.36)**	− **0.81 (**− **1.30, **− **0.33)**	− **0.55 (**− **1.03, **− **0.07)**
Change	3537	0.46 (− 0.18, 1.09)	− 0.17 (− 0.66, 0.33)	− 0.15 (− 0.64, 0.34)
Digit span^c^				
CRAE				
Exam 2	4142	− 0.05 (− 0.19, 0.08)	0.04 (− 0.08, 0.16)	0.02 (− 0.10, 0.15)
Exam 5	3964	− **0.24 (**− **0.38, **− **0.10)**	− 0.07 (− 0.20, 0.06)	− 0.07 (− 0.20, 0.06)
Change	3804	− **0.29 (**− **0.45, **− **0.13)**	− 0.13 (− 0.27, 0.01)	− 0.11 (− 0.24, 0.03)
CRVE				
Exam 2	4158	− **0.26 (**− **0.40, **− **0.12)**	− 0.04 (− 0.17, 0.09)	− 0.01 (− 0.14, 0.12)
Exam 5	3994	− **0.23 (**− **0.38, **− **0.09)**	− 0.02 (− 0.15, 0.11)	− 0.02 (− 0.15, 0.11)
Change	3841	− 0.04 (− 0.19, 0.11)	0.03 (− 0.11, 0.16)	0.01 (− 0.13, 0.14)

Significant values are in [bold].

Model 1 adjustments include age, sex, race, and education (completed high school or more vs. did not complete high school).

Model 2 included Model 1 adjustments plus body mass index, hypertension, diabetes, cigarette smoking, current alcohol use, low-density lipoproteins (optimal vs. non-optimal), c-reactive protein, albuminuria, and incident stroke between Exam 2 and Exam 5.

*CASI* Cognitive Abilities Screening Instrument; *CRAE* central retinal artery equivalent; *CRVE* central retinal vein equivalent.

^a^n = 4334 with valid Exam 5 CASI and at least one retinal vessel caliber measure.

^b^n = 3957 with valid Exam 5 Digit Symbol Coding test and at least one retinal vessel caliber measure.

^c^n = 4320 with valid Exam 5 Digit Span test and at least one retinal vessel caliber measure.

**Table 3:** Associations of mean retinal caliber with change in cognitive tests (CASI, Digit Symbol Coding, Digit Span) performance over 6 years among participants with repeat cognitive testing at Exam 6.

	n	Score trajectory per SD increment in retinal caliber (95% CI)^a^
CASI^a^		
CRAE		
Exam 2	1778	− 0.03 (− 0.33, 0.27)
Exam 5	1729	− 0.03 (− 0.33, 0.27)
Change	1681	0.09 (− 0.24, 0.42)
CRVE		
Exam 2	1785	− 0.08 (− 0.35, 0.19)
Exam 5	1735	0.11 (− 0.21, 0.42)
Change	1692	**0.33 (0.01, 0.66)**
Digit symbol coding^b^		
CRAE		
Exam 2	1439	0.03 (− 0.54, 0.60)
Exam 5	1398	0.06 (− 0.51, 0.62)
Change	1363	− 0.30 (− 0.92, 0.32)
CRVE		
Exam 2	1443	0.34 (− 0.26, 0.93)
Exam 5	1403	0.36 (− 0.22, 0.94)
Change	1371	0.04 (− 0.57, 0.66)
Digit span^c^		
CRAE		
Exam 2	1723	0.04 (− 0.10, 0.18)
Exam 5	1669	− 0.001 (− 0.14, 0.14)
Change	1625	− 0.06 (− 0.21, 0.10)
CRVE		
Exam 2	1727	− 0.07 (− 0.21, 0.07)
Exam 5	1676	− 0.03 (− 0.17, 0.11)
Change	1635	− 0.01 (− 0.16, 0.14)

Significant values are in [bold].

^a^Adjusted for Model 2 covariates (age, sex, race/ethnicity, educational attainment, body mass index, hypertension, diabetes, cigarette smoking, current alcohol use, low-density lipoproteins [optimal vs. non-optimal], c-reactive protein, albuminuria, and incident stroke between Exam 2 and Exam 5) and Exam 5 cognitive test score.

^a^n = 1831 with repeat CASI data at Exam 6 and at least one retinal vessel caliber measure.

^b^n = 1478 with repeat Digit Symbol Coding test data at Exam 6 and at least one retinal vessel caliber measure.

^c^n = 1772 with repeat Digit Span test data at Exam 6 and at least one retinal vessel caliber measure.

### Performance on speed of processing and working memory tests

In fully adjusted models, larger CRVE at Exam 2 and Exam 5 was associated with worse Digit Symbol Coding performance (− 0.56 [95% CI − 1.02, − 0.09] and − 0.55 [95% CI − 1.03, − 0.07] points respectively; Table 2). Associations of CRAE and CRVE with the Digit Symbol Coding test differed by sex (Exam 2 CRAE interaction *p* = 0.025; Exam 5 CRAE interaction *p* = 0.007; Exam 2 CRVE interaction *p* = 0.030). Statistically significant associations of CRVE with Digit Symbol Coding performance were driven more strongly by men (Supplemental Table 3). There were no associations of retinal calibers with the Digit Span test (Table 2), or with change in Digit Symbol Coding or Digit Span performance from Exam 5 to Exam 6. Results did not differ by race/ethnicity for any cognitive test.

## Discussion

In this large, ethnically and racially diverse cohort, after adjustment for multiple demographic and cardiovascular risk factors, wider retinal venular calibers were associated with slower concomitant and future processing speed however not with later cognitive trajectories.

The interaction by sex was significant in the association of CRVE with Digit Symbol Coding wherein the association was driven strongly by men. It is possible that this finding is due to chance or it could be due to sex-related differences in cardiovascular risk factors and its sequelae^24^. For example, in a retinal photograph-based deep learning model, compared to women, retinal characteristics better stratified biological age in men^25^. Similar to other large cohorts, men at baseline in MESA had a worse cardiovascular risk profile and a higher ASCVD risk score (17%) when compared to women (12%), *p* < 0.001. Men also had poorer cognitive performance at baseline: Digit Symbol Coding was lower in men as compared to women (50.1 vs. 51.6, *p* = 0.008). Baseline CRVE, however, did not differ between men and women. Future studies evaluating the direction of the association by sex are warranted.

### Retinal vascular caliber and cardiovascular risk factors

Retinal vascular caliber may be an early vascular indicator related to cognitive function by reflecting microvascular disease possibly associated with cerebrovascular disease or through its association with other cardiovascular risk factors.

While other unmeasured confounders may affect vascular caliber such as refractive error, the association of retinal arteriolar diameters with cardiovascular risk factors such as hypertension tends to be minimally affected by refractive error^17^. Previous studies have demonstrated multiple associations of retinal vascular characteristics and retinopathy with incident stroke and indicators of small vessel disease on neuroimaging^26^. For example, in MESA after adjustment for conventional risk factors, a lower retinal arteriolar caliber was associated with a threefold increased risk of stroke^27^. Retinal vascular calibers have been variably associated with other cardiovascular risk factors: for example, a narrower arteriolar caliber and wider venular caliber are associated with hypertension^28,29^. In the United Kingdom (UK) Biobank cohort study, narrower arterioles were associated with higher blood pressure and arterial stiffness index^30^. A weaker association of retinal arteriolar diameters and blood pressure in older people may reflect greater sclerosis of the retinal arterioles, preventing a degree of narrowing with higher blood pressure similar to that seen in younger persons^31^. In contrast, in a cohort of participants with diabetes, after adjusting for vascular risk factors, larger CRAE and CRVE were associated with diabetic retinopathy^32^. Others showed that a lower CRAE is more likely found among those with higher systolic blood pressure, increased age, and higher HDL cholesterol whereas a larger CRAE and CRVE are more common among those who smoke^33^. In the ARIC cohort, the major systemic determinant of lower retinal arterial caliber was higher blood pressure, while those of wider retinal vein caliber were cigarette smoking, higher blood pressure, systemic inflammation, and obesity^34^. In the UK biobank, fractal dimension [FD], a measure of the complexity of the vascular network, rather than retinal arteriolar and venular caliber were associated with albuminuria^35^. Using large-scale complementary machine learning-based assessment of the retinal vasculature, low retinal vascular fractal dimension and density were significantly associated with incident mortality as well as hypertension, congestive heart failure, renal failure, type 2 diabetes and sleep apnea^36^. In the UK Biobank, artificial intelligence (AI)-enabled retinal vasculometry that included retinal arteriolar and venular width, tortuosity and area, offered an alternative predictive biomarker to traditional risk-scores for vascular health suggesting a potential utility of retinal vasculometry in population level prediction of cardiovascular risk^37^. Because of the effect of cardiovascular risk factors on the retinal vasculature and the potential effect of microvascular and macrovascular risk factors on cognition, we included vascular risk factors in adjustment models.

### Cross-sectional studies

We found that a larger CRVE at exam 5 was associated with slower speed of processing (Digit Symbol Coding), an association that remained significant after adjustment for demographic and cardiovascular risk factors. Similarly, we found that a larger CRAE was associated with a lower global cognitive function (CASI) when both were measured during the same time period. As discussed in the previous section, CRAE may be affected by multiple cardiovascular risk factors, and is usually higher with diabetes and cigarette smoking and lower with hypertension. The effect of hypertension on CRAE tends to be lower with older age. These considerations, as well as other potential unmeasured confounders may explain the unexpected finding that a larger CRAE (rather than a lower CRAE) was weakly associated with a lower CASI. Several cross-sectional studies had previously evaluated the association of retinal microvascular caliber with cognitive performance and found differing results^3^. Retinopathy rather than microvascular caliber was found to be associated with cognitive impairment and more pronounced in the setting of hypertension but results were not consistent likely due to the heterogeneity of the cohort characteristics and study designs^3^. Associations with retinal vascular calibers were found in both directions and were mostly not statistically significant^3^. In one study of 809 elderly Latino participants in the Los Angeles Latino Eye Study (LALES), the association of retinal caliber and cognitive performance was driven by participants with hypertension and only when the cognitive outcome was dichotomized^38^. Similarly, CRVE and CRAE were not associated with cognitive function in the Northern Ireland Cohort for the Longitudinal Study of Aging^39^. As compared to cognitively normal controls, a sparser microvascular network, rather than the venular or arteriolar caliber (CRAE or CRVE), was associated with the diagnosis of Alzheimer’s disease^40^. In the current study, we analyzed cognitive performance as a continuous variable because cognitive status (cognitive impairment vs. not) had not been adjudicated. The difference between the MESA cohort demographic and comorbidities characteristics with other cohorts included in reported studies may also explain some of the differing results.

### Longitudinal studies

Our study is interesting because in addition to the cross-sectional data in a large and diverse cohort free from dementia at baseline, it provides longitudinal data with both repeated retinal caliber measurements and subsequent repeated cognitive function.

CRVE, CRAE and their change over time were normally distributed and relatively stable with CRVE showing a slightly larger change compared to CRAE over the 8 years’ period of the study. This is consistent with the limited previous literature on longitudinal changes with repeated retinal measures. In a study of healthy children aged 7–9 years followed over a period of 5 years, the fluctuation in CRVE was slightly higher than that of CRAE and associated with BMI^41^. Similarly, in a cohort of adults with diabetes followed over 4 years, the mean change in CRVE was larger than the change in CRAE and was associated with the incidence and progression of diabetic retinopathy^42^.

In contrast to cross sectional studies, longitudinal studies investigating the association of CRVE and CRAE with cognitive outcomes are less common, and have heterogeneous study designs and outcome measures^3^. A 2013 meta-analysis found a paucity of longitudinal studies and suggested the need for additional longitudinal data from large datasets^3^. For example, in ARIC, individuals with retinopathy at baseline (retinal microaneurysms or blot hemorrhages) and focal retinal arteriolar narrowing showed decline in executive function and psychomotor speed over 14 years^10^. However, neither the lowest quartile of arterial diameter nor the largest quartile of vein diameter was associated with cognitive decline. Arterial and vein diameters also failed to show significant associations with cognitive decline when they were analyzed as continuous variables. Because retinal characteristics in the ARIC study were only measured at baseline (in 1993–1995), changes in CRAE and CRVE were not available for analysis^10^. In a study of deep-learning algorithm using retinal vessel caliber measurements, narrower retinal arteriolar caliber and wider venular calibers at baseline were associated with an increased risk of cognitive decline^43^.

In contrast, fewer studies have evaluated the trajectory of CRAE and CRVE in relation to cognitive function. For example, in the Pittsburgh Epidemiology of Diabetes Complications Study which included participants with type 1 diabetes (mean age 43 years), participants with clinically relevant cognitive impairment experienced 1.8% greater and 31.1% faster CRAE narrowing during prior years (between the baseline measurements in 1986–1988 and follow-up in 2010–2015) compared with participants without cognitive impairment. No associations were found between central retinal arterial equivalent or central retinal vein equivalent measures at baseline or at time of cognitive testing and cognitive impairment^28^.

In the current analysis, in the fully adjusted models, the CRVE on Exam 2 was associated with both the CASI and the Digit Symbol Coding on Exam 5 (mean follow-up of 8 years). Retinal vein widening has been found previously to be associated with incident dementia in the Rotterdam study^28,44^. In a study of 251 children aged 4 to 5 years, retinal venular widening and a higher vessel tortuosity were associated with a lower performance of short-term visual recognition memory^45^. Some studies suggest that venular widening may be a marker of other processes, such as endothelial dysfunction, hypoperfusion, and cerebral hypoxia^18,46^. Previous studies have suggested a role of venules in the development of white matter disease. Specifically, venous collagenosis dilates the veins causing venous insufficiency with consequent vessel leakage that may lead to nonnecrotic hyperintensities seen in the spectrum of white matter disease^47^. Because brain imaging was not available at the time of this study, it is not possible to determine whether the association of dilated retinal veins with cognition is mediated by a higher prevalence of white matter disease, but it does raise an interesting hypothesis. A subsequent MESA study found greater arteriolar fractal dimension to be associated with MRI biomarkers indicative of less neuroinflammation and neurodegeneration^48^.

It is interesting that the trajectory of venous caliber in the preceding 8 years was not associated with cognitive function suggesting that retinal venular widening may reflect the effect of a stronger mediating factor or that baseline retinal widening, rather than the trajectory of change, is an indicator of neuropsychological health. Further supporting this hypothesis is the finding that an increase in CRVE over time was associated with a future slight improvement in CASI instead of a decline in CASI. This association was small, does not have a clear biological basis and is not likely to be clinically significant but it does further suggest that the trajectory of CRVE change over time is less indicative of cognitive function than the baseline CRVE. Other studies have suggested that venular caliber may be determined early in life and associated with overall neuropsychological functioning. For example, in the Dunedin birth cohort, a wider venular caliber was associated with poor neuropsychological functioning at midlife and with lower childhood IQ tested 25 years earlier^49^.

### Limitations

This study has several limitations. Despite multiple statistical adjustments and the implementation of inverse probability weighting, it is possible that these results may still be biased by additional unmeasured confounding or from differential missingness in the data. Neuroimaging data were not available at this time in MESA but could have been useful to evaluate whether observed relationships between retinal caliber and cognitive performance were indicative of microvascular disease on MRI such as white matter disease or cerebral microhemorrhages. Clinical cognitive status (cognitively intact vs. cognitive impairment) was not adjudicated in this sample. Since cognitive function was not formally assessed at baseline, we cannot completely rule out the possibility that some participants entered MESA with cognitive impairment. However, individuals with evidence of clinically recognized dementia (self-reported) were excluded from participation in MESA. Because excluded participants tended to be less healthy and have lower cognitive scores, a different or more significant association may have been found in sicker or more cognitively impaired individuals. This is a cohort of older adults, and it is possible that CRVE and CRAE are influenced by factors across the lifespan which may, in turn, impact on an association with cognition differently in other age groups. However, participants were relatively young for age-related cognitive decline and dementing illnesses. Despite these limitations, we considered multiple relevant vascular risk factors and evaluated for relevant interactions. We also combined inverse probability weighting and multivariate imputation methods in our analysis to minimize bias resulting from exclusion, loss to follow-up, and missing data.

## Conclusions

Larger retinal venular calibers were associated with a lower concomitant and subsequent scores on processing speed testing and a lower subsequent score of global cognitive function but not with later cognitive trajectory. These associations were more significant in men. Additional studies are needed to evaluate the utility of these measures in risk stratification models for clinical care as well as their utility for screening on a population level.

### Supplementary Information

Supplementary Information.

## Data Availability

The data that support the findings of this study are available from the MESA Coordinating Center. The authors are restricted from further distribution of the data. Interested investigators can request and access MESA deidentified datasets (with HIPAA defined identifiers removed) by completing a Data Distribution Agreement that will be reviewed for approval by the MESA Publications and Steering Committees. The data request is accessible via the following website: https://biolincc.nhlbi.nih.gov/studies/mesa/.

## References

[1] Gorelick PB, Scuteri A, Black SE (2011). Vascular contributions to cognitive impairment and dementia: A statement for healthcare professionals from the American heart association/American stroke association. Stroke.

[2] Cheung CY, Chan VTT, Mok VC, Chen C, Wong TY (2019). Potential retinal biomarkers for dementia: What is new?. Curr Opin Neurol.

[3] Heringa SM, Bouvy WH, van den Berg E, Moll AC, Kappelle LJ, Biessels GJ (2013). Associations between retinal microvascular changes and dementia, cognitive functioning, and brain imaging abnormalities: A systematic review. J Cereb Blood Flow Metab.

[4] Sharrett AR (2007). A review of population-based retinal studies of the microvascular contribution to cerebrovascular diseases. Ophthalmic Epidemiol.

[5] Ding J, Patton N, Deary IJ (2008). Retinal microvascular abnormalities and cognitive dysfunction: A systematic review. Br J Ophthalmol.

[6] Wong TY, McIntosh R (2005). Systemic associations of retinal microvascular signs: A review of recent population-based studies. Ophthalmic Physiol Opt.

[7] Wong TY, Mitchell P (2007). The eye in hypertension. Lancet.

[8] Ong YT, Hilal S, Cheung CY (2014). Retinal vascular fractals and cognitive impairment. Dement Geriatr Cogn Dis Extra.

[9] Cheung CY, Mok V, Foster PJ, Trucco E, Chen C, Wong TY (2021). Retinal imaging in Alzheimer's disease. J Neurol Neurosurg Psychiatry.

[10] Lesage SR, Mosley TH, Wong TY (2009). Retinal microvascular abnormalities and cognitive decline: The ARIC 14-year follow-up study. Neurology.

[11] Bild DE, Bluemke DA, Burke GL (2002). Multi-Ethnic Study of Atherosclerosis: Objectives and design. Am J Epidemiol.

[12] Bertoni AG, Burke GL, Owusu JA (2010). Inflammation and the incidence of type 2 diabetes: The Multi-Ethnic Study of Atherosclerosis (MESA). Diabetes Care.

[13] Kramer H, Jacobs DR, Bild D (2005). Urine albumin excretion and subclinical cardiovascular disease. The Multi-Ethnic Study of Atherosclerosis. Hypertension.

[14] Wong TY, Klein R, Islam FM (2006). Diabetic retinopathy in a multi-ethnic cohort in the United States. Am J Ophthalmol.

[15] Klein R, Klein BE, Knudtson MD (2006). Prevalence of age-related macular degeneration in 4 racial/ethnic groups in the multi-ethnic study of atherosclerosis. Ophthalmology.

[16] Hubbard LD, Brothers RJ, King WN (1999). Methods for evaluation of retinal microvascular abnormalities associated with hypertension/sclerosis in the Atherosclerosis Risk in Communities Study. Ophthalmology.

[17] Wong TY, Knudtson MD, Klein R, Klein BE, Meuer SM, Hubbard LD (2004). Computer-assisted measurement of retinal vessel diameters in the Beaver Dam Eye Study: Methodology, correlation between eyes, and effect of refractive errors. Ophthalmology.

[18] Wong TY, Islam FM, Klein R (2006). Retinal vascular caliber, cardiovascular risk factors, and inflammation: The multi-ethnic study of atherosclerosis (MESA). Invest Ophthalmol Vis Sci.

[19] Knudtson MD, Lee KE, Hubbard LD, Wong TY, Klein R, Klein BE (2003). Revised formulas for summarizing retinal vessel diameters. Curr Eye Res.

[20] Fitzpatrick AL, Rapp SR, Luchsinger J (2015). Sociodemographic correlates of cognition in the multi-ethnic study of atherosclerosis (MESA). Am J Geriatr Psychiatry.

[21] Wechsler D (1996). Wechsler Adult Intelligence Scale-III (WAIS-III).

[22] Hughes TM, Craft S, Baker LD (2017). Changes in metabolic risk factors over 10 years and their associations with late-life cognitive performance: The Multi-Ethnic Study of Atherosclerosis. Alzheimers Dement (Amst).

[23] Avery CL, Kucharska-Newton A, Monda KL (2012). Impact of long-term measures of glucose and blood pressure on the retinal microvasculature. Atherosclerosis.

[24] Volgman AS, Bairey Merz CN, Aggarwal NT (2019). Sex differences in cardiovascular disease and cognitive impairment: Another health disparity for women?. J Am Heart Assoc.

[25] Nusinovici S, Rim TH, Yu M (2022). Retinal photograph-based deep learning predicts biological age, and stratifies morbidity and mortality risk. Age Ageing.

[26] Hughes AD, Falaschetti E, Witt N (2016). Association of retinopathy and retinal microvascular abnormalities with stroke and cerebrovascular disease. Stroke.

[27] Kawasaki R, Xie J, Cheung N (2012). Retinal microvascular signs and risk of stroke: The Multi-Ethnic Study of Atherosclerosis (MESA). Stroke.

[28] Nunley KA, Metti AL, Klein R (2018). Long-term changes in retinal vascular diameter and cognitive impairment in type 1 diabetes. Diab Vasc Dis Res.

[29] Kawasaki R, Cheung N, Wang JJ (2009). Retinal vessel diameters and risk of hypertension: The Multiethnic Study of Atherosclerosis. J Hypertens.

[30] Tapp RJ, Owen CG, Barman SA (2019). Associations of retinal microvascular diameters and tortuosity with blood pressure and arterial stiffness: United Kingdom biobank. Hypertension.

[31] Wong TY, Klein R, Klein BE, Meuer SM, Hubbard LD (2003). Retinal vessel diameters and their associations with age and blood pressure. Invest Ophthalmol Vis Sci.

[32] Drobnjak D, Munch IC, Glumer C (2017). Relationship between retinal vessel diameters and retinopathy in the Inter99 Eye Study. J Clin Transl Endocrinol.

[33] Drobnjak D, Munch IC, Glumer C (2016). Retinal vessel diameters and their relationship with cardiovascular risk and all-cause mortality in the inter99 eye study: A 15-year follow-up. J Ophthalmol.

[34] Liew G, Sharrett AR, Wang JJ (2008). Relative importance of systemic determinants of retinal arteriolar and venular caliber: The atherosclerosis risk in communities study. Arch Ophthalmol.

[35] Paterson EN, Cardwell C, MacGillivray TJ (2021). Investigation of associations between retinal microvascular parameters and albuminuria in UK Biobank: A cross-sectional case-control study. BMC Nephrol.

[36] Zekavat SM, Raghu VK, Trinder M (2022). Deep learning of the retina enables phenome- and genome-wide analyses of the microvasculature. Circulation.

[37] Rudnicka AR, Welikala R, Barman S (2022). Artificial intelligence-enabled retinal vasculometry for prediction of circulatory mortality, myocardial infarction and stroke. Br J Ophthalmol.

[38] Gatto NM, Varma R, Torres M (2012). Retinal microvascular abnormalities and cognitive function in Latino adults in Los Angeles. Ophthalmic Epidemiol.

[39] O'Neill RA, Maxwell AP, Paterson EN (2021). Retinal microvascular parameters are not significantly associated with mild cognitive impairment in the Northern Ireland Cohort for the Longitudinal Study of Ageing. BMC Neurol.

[40] Williams MA, McGowan AJ, Cardwell CR (2015). Retinal microvascular network attenuation in Alzheimer's disease. Alzheimers Dement (Amst).

[41] Kurniawan ED, Cheung CY, Tay WT (2014). The relationship between changes in body mass index and retinal vascular caliber in children. J Pediatr.

[42] Klein R, Myers CE, Lee KE, Gangnon R, Klein BE (2012). Changes in retinal vessel diameter and incidence and progression of diabetic retinopathy. Arch Ophthalmol.

[43] Cheung CY, Wong WLE, Hilal S (2022). Deep-learning retinal vessel calibre measurements and risk of cognitive decline and dementia. Brain Commun.

[44] de Jong FJ, Schrijvers EM, Ikram MK (2011). Retinal vascular caliber and risk of dementia: The Rotterdam study. Neurology.

[45] Luyten LJ, Dockx Y, Madhloum N (2020). Association of retinal microvascular characteristics with short-term memory performance in children aged 4 to 5 years. JAMA Netw Open.

[46] de Jong FJ, Vernooij MW, Ikram MK (2008). Arteriolar oxygen saturation, cerebral blood flow, and retinal vessel diameters. The Rotterdam Study. Ophthalmology.

[47] Black S, Gao F, Bilbao J (2009). Understanding white matter disease: Imaging-pathological correlations in vascular cognitive impairment. Stroke.

[48] Ong, S.S., Peavey, J.J. & Hiatt, K.D., et al. Association of fractal dimension and other retinal vascular network parameters with cognitive performance and neuroimaging biomarkers: The Multi-Ethnic Study of Atherosclerosis (MESA). Alzheimers Dement 2023. 10.1002/alz.13498 PMC10916935 37828734

[49] Shalev I, Moffitt TE, Wong TY (2013). Retinal vessel caliber and lifelong neuropsychological functioning: Retinal imaging as an investigative tool for cognitive epidemiology. Psychol Sci.

