# Supplementary material for: Retinal vessel caliber and cognitive performance: the multi-ethnic study of atherosclerosis (MESA)
Source: Sci Rep. 2024 Feb 19;14:4120. doi: 10.1038/s41598-024-54412-2 (PMC10876697; doi:10.1038/s41598-024-54412-2)
Supplement: Supplementary file 1 — Supplementary Information. [file 41598_2024_54412_MOESM1_ESM.docx]

**Supplemental Tables**

| **Supplemental Table 1.** Standardized Weighted and Unweighted Mean Differences between Participants Missing and Not Missing Retinal Caliber Measures. | | |
| --- | --- | --- |
|  | **Weighted** | **Unweighted** |
| **Age, Exam 2, years** | 0.124 | 0.676 |
| **CRP, Exam 1, mg/L** | 0.005 | 0.022 |
| **BMI, Exam 2, kg/m^2^** | 0.034 | 0.111 |
| **BMI, Exam 5, kg/m^2^** | 0.010 | 0.024 |
| **CASI, Exam 5** | 0.089 | 0.415 |
| **Digit Symbol Coding, Exam 5** | 0.085 | 0.533 |
| **Digit Span, Exam 5** | 0.011 | 0.198 |
| **Column mean** | 0.014 | 0.045 |
| BMI, body mass index; CASI, Cognitive Abilities Screening Instrument; CRP, c-reactive protein. | | |
| *Note:* Standardized mean differences are calculated as the absolute difference in means between participants missing and not missing retinal caliber data, divided by the average of the within-group standard deviations. Weighted differences closer to 0 than unweighted differences indicate better balance achieved by inverse probability weighting. | | |

| **Supplemental Table 2.** Standardized Weighted and Unweighted Mean Differences between Participants Missing and Not Missing Repeat CASI, Digit Symbol Coding, or Digit Span Data. | | | | | | |
| --- | --- | --- | --- | --- | --- | --- |
|  | **CASI** | | **Digit Symbol Coding** | | **Digit Span** | |
|  | **Weighted** | **Unweighted** | **Weighted** | **Unweighted** | **Weighted** | **Unweighted** |
| **Age, Exam 2, years** | 0.230 | 0.430 | 0.022 | 0.397 | 0.015 | 0.376 |
| **CRP, Exam 1, mg/L** | 0.019 | 0.048 | 0.028 | 0.048 | 0.028 | 0.048 |
| **BMI, Exam 2, kg/m^2^** | 0.006 | 0.010 | 0.009 | 0.026 | 0.007 | 0.024 |
| **BMI, Exam 5, kg/m^2^** | 0.010 | 0.060 | 0.007 | 0.016 | 0.014 | 0.019 |
| **CASI, Exam 5** | 0.187 | 0.418 | 0.216 | 0.398 | 0.193 | 0.376 |
| **Digit Symbol Coding, Exam 5** | 0.133 | 0.430 | 0.177 | 0.428 | 0.170 | 0.403 |
| **Digit Span, Exam 5** | 0.050 | 0.224 | 0.055 | 0.209 | 0.040 | 0.190 |
| **CRAE, Exam 2** | 0.010 | 0.029 | 0.015 | 0.024 | 0.015 | 0.021 |
| **CRAE, Exam 5** | 0.016 | 0.017 | 0.001 | 0.019 | 0.007 | 0.025 |
| **CRVE, Exam 2** | 0.031 | 0.031 | 0.021 | 0.025 | 0.013 | 0.023 |
| **CRVE, Exam 5** | 0.005 | 0.041 | 0.009 | 0.022 | 0.001 | 0.018 |
| **Column means** | 0.063 | 0.158 | 0.051 | 0.147 | 0.046 | 0.138 |
| BMI, body mass index; CASI, Cognitive Abilities Screening Instrument; CRAE, central retinal artery equivalent; CRP, c-reactive protein; CRVE, central retinal vein equivalent. | | | | | | |
| *Note:* Standardized mean differences are calculated as the absolute difference in means between participants missing and not missing respective repeat cognitive data at Exam 6, divided by the average of the within-group standard deviations. Weighted differences closer to 0 than unweighted differences indicate better balance achieved by inverse probability weighting. | | | | | | |

| **Supplemental Table 3.** Association of retinal calibers with Digit Symbol Coding performance in men and women. | | |
| --- | --- | --- |
|  | **Difference in test score per SD increment in retinal caliber (95% CI)^a^** | |
|  | **Men** | **Women** |
| **Digit Symbol Coding** |  |  |
| CRAE |  |  |
| Exam 2 | -0.51 (-1.17, 0.16) | 0.38 (-0.26, 1.01) |
| Exam 5 | -0.51 (-1.18, 0.16) | 0.64 (-0.01, 1.29) |
| Change | 0.05 (-0.59, 0.69) | 0.18 (-0.47, 0.83) |
| CRVE |  |  |
| Exam 2 | **-1.09 (-1.78, -0.40)** | -0.13 (-0.78, 0.52) |
| Exam 5 | **-0.81 (-1.50, -0.12)** | -0.31 (-0.98, 0.37) |
| Change | 0.15 (-0.51, 0.80) | 0.05 (-0.58, 0.69) |
| ^a^Adjusted for Model 2 covariates (age, race/ethnicity, educational attainment, body mass index, hypertension, diabetes, cigarette smoking, current alcohol use, low-density lipoproteins [optimal vs. non-optimal], c-reactive protein, albuminuria, and incident stroke between Exam 2 and Exam 5). | | |

**Supplemental Figure 1.**

**
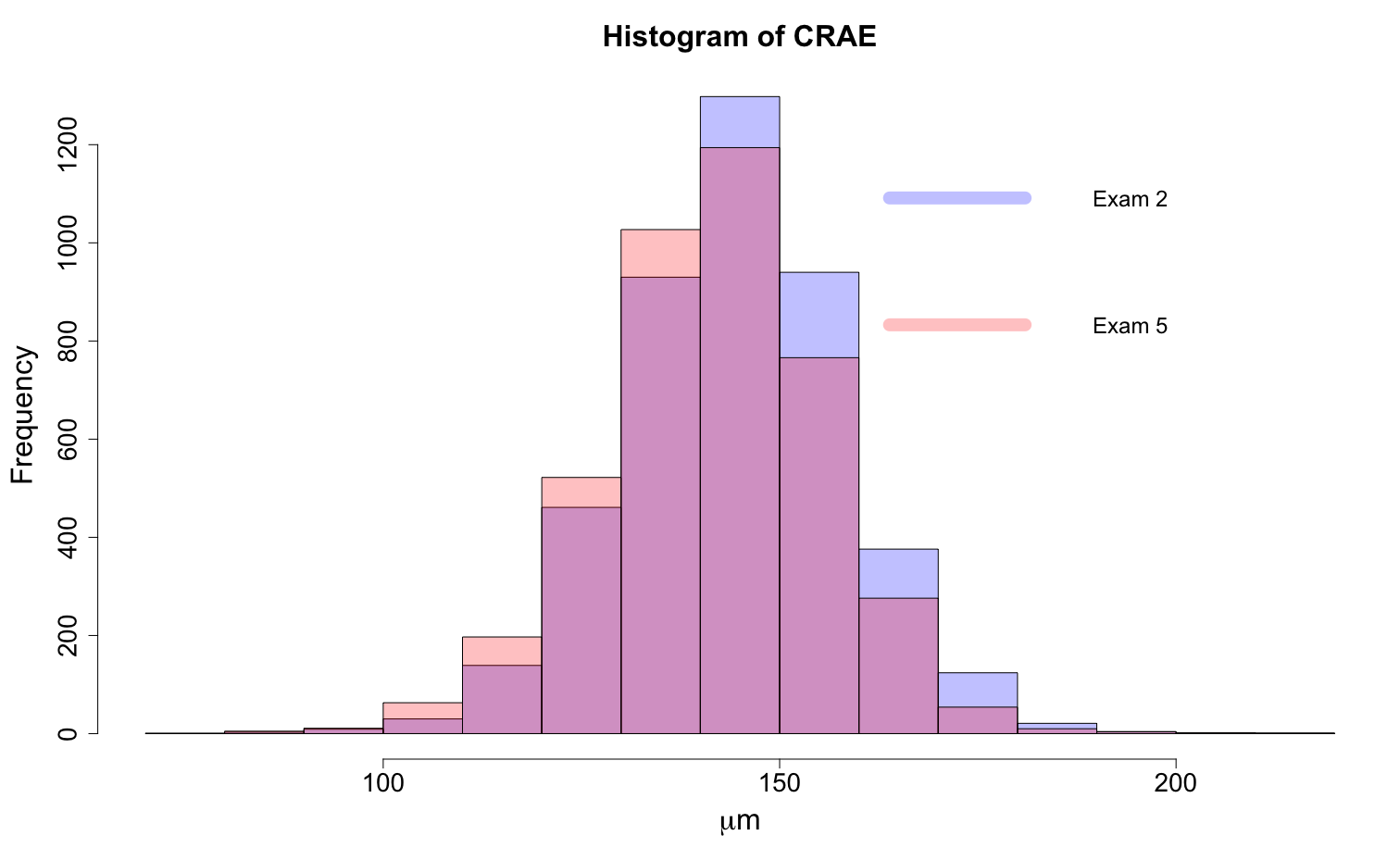
**

> Summary statistics for Exam 2 CRAE (µm)

Min. 1st Qu. Median Mean 3rd Qu. Max. SD

78.93 135.09 144.57 144.30 153.39 219.13 14.0

> Summary statistics for Exam 5 CRAE (µm)

Min. 1st Qu. Median Mean 3rd Qu. Max. SD

80.5 132.8 141.9 141.5 150.7 213.9 14.1

**Supplemental Figure 2.**


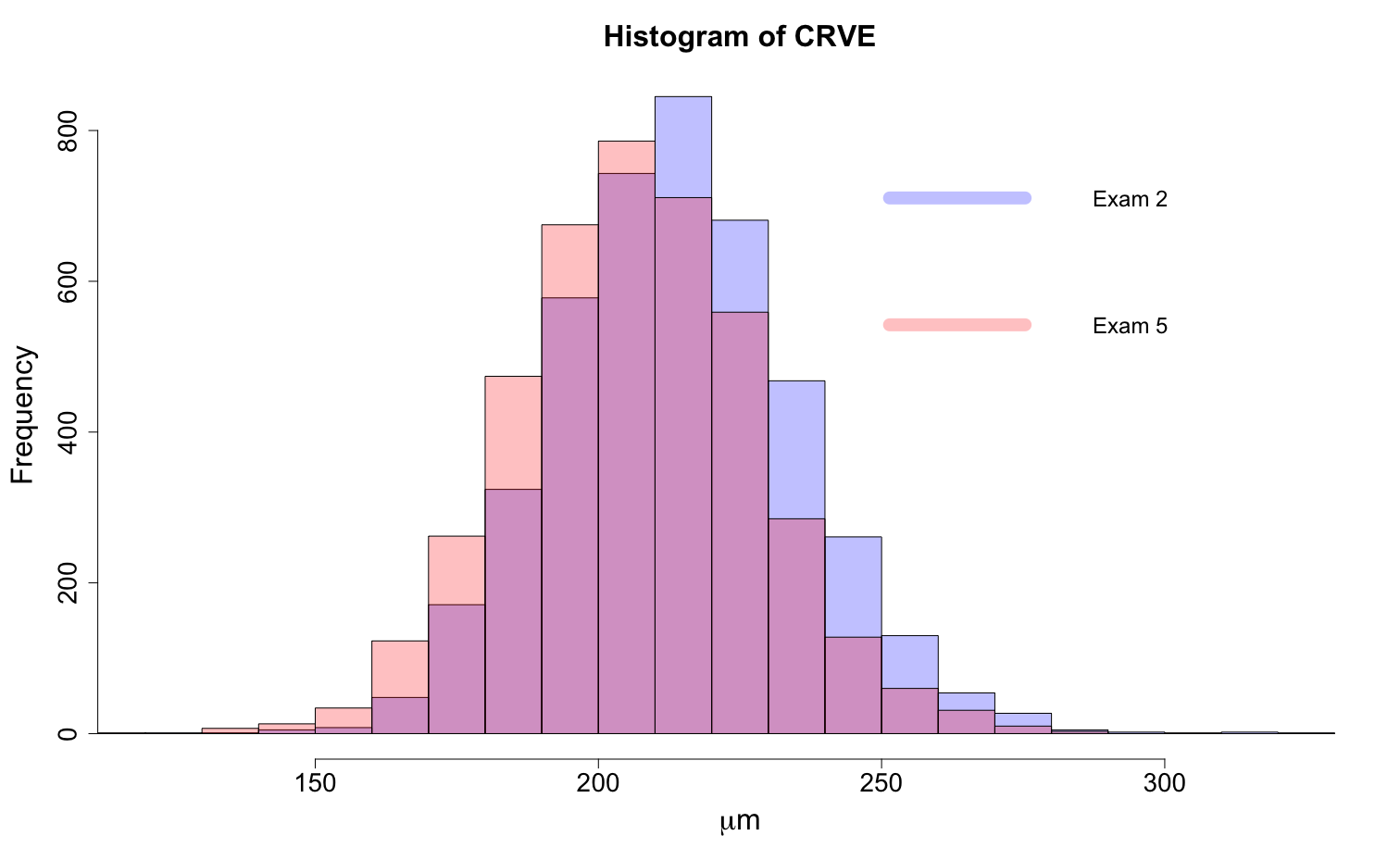


> Summary statistics for Exam 2 CRVE (µm)

Min. 1st Qu. Median Mean 3rd Qu. Max. SD

123.6 199.3 213.6 214.0 227.9 323.9 21.6

> Summary statistics for Exam 5 CRVE (µm)

Min. 1st Qu. Median Mean 3rd Qu. Max. SD

117.7 192.1 206.3 206.4 220.5 305.6 21.5

**Supplemental Figure 3.**


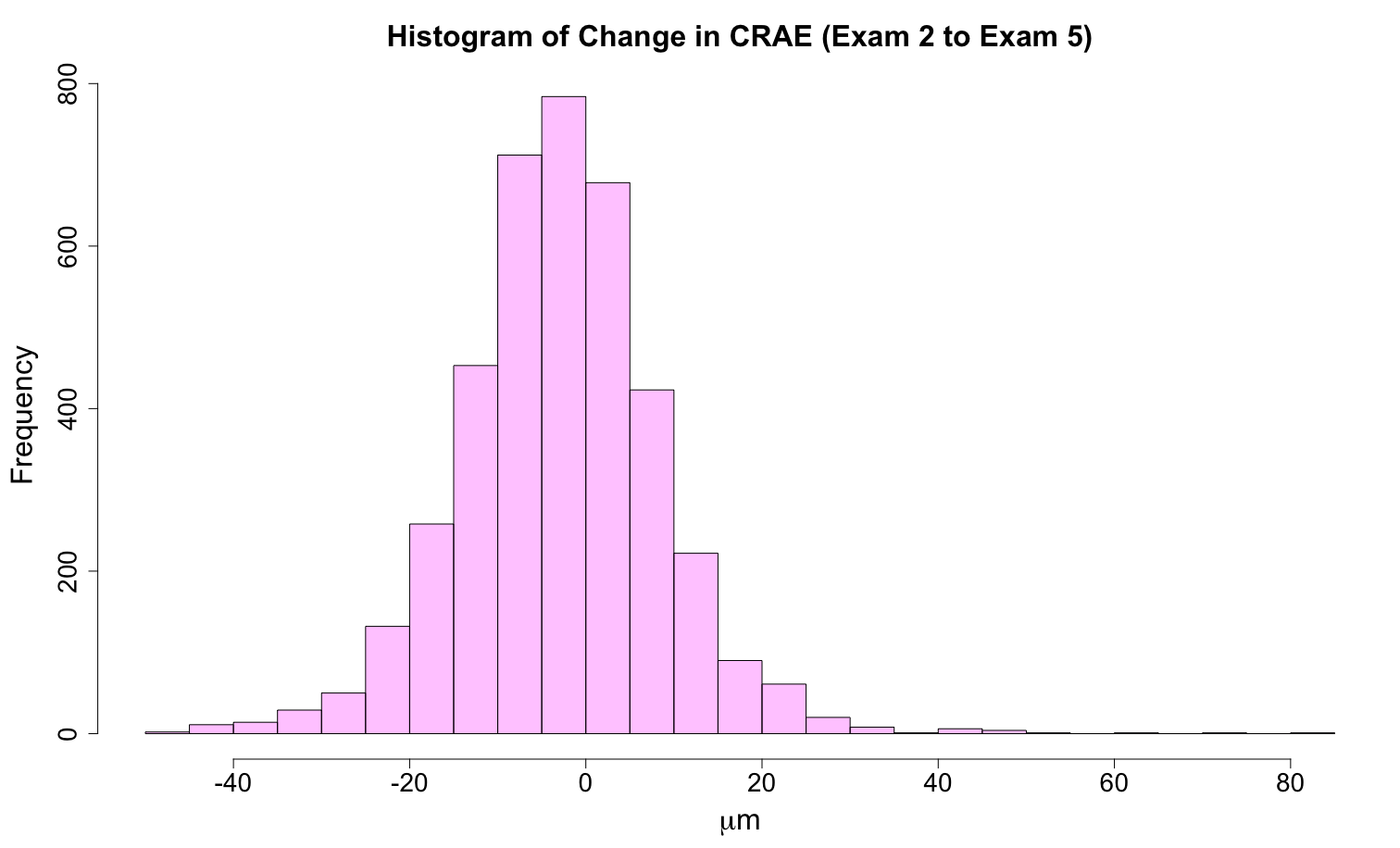


> Summary statistics for Change in CRAE (µm)

Min. 1st Qu. Median Mean 3rd Qu. Max. SD

-46.522 -9.649 -3.055 -2.954 3.705 82.285 11.4

**Supplemental Figure 4.**


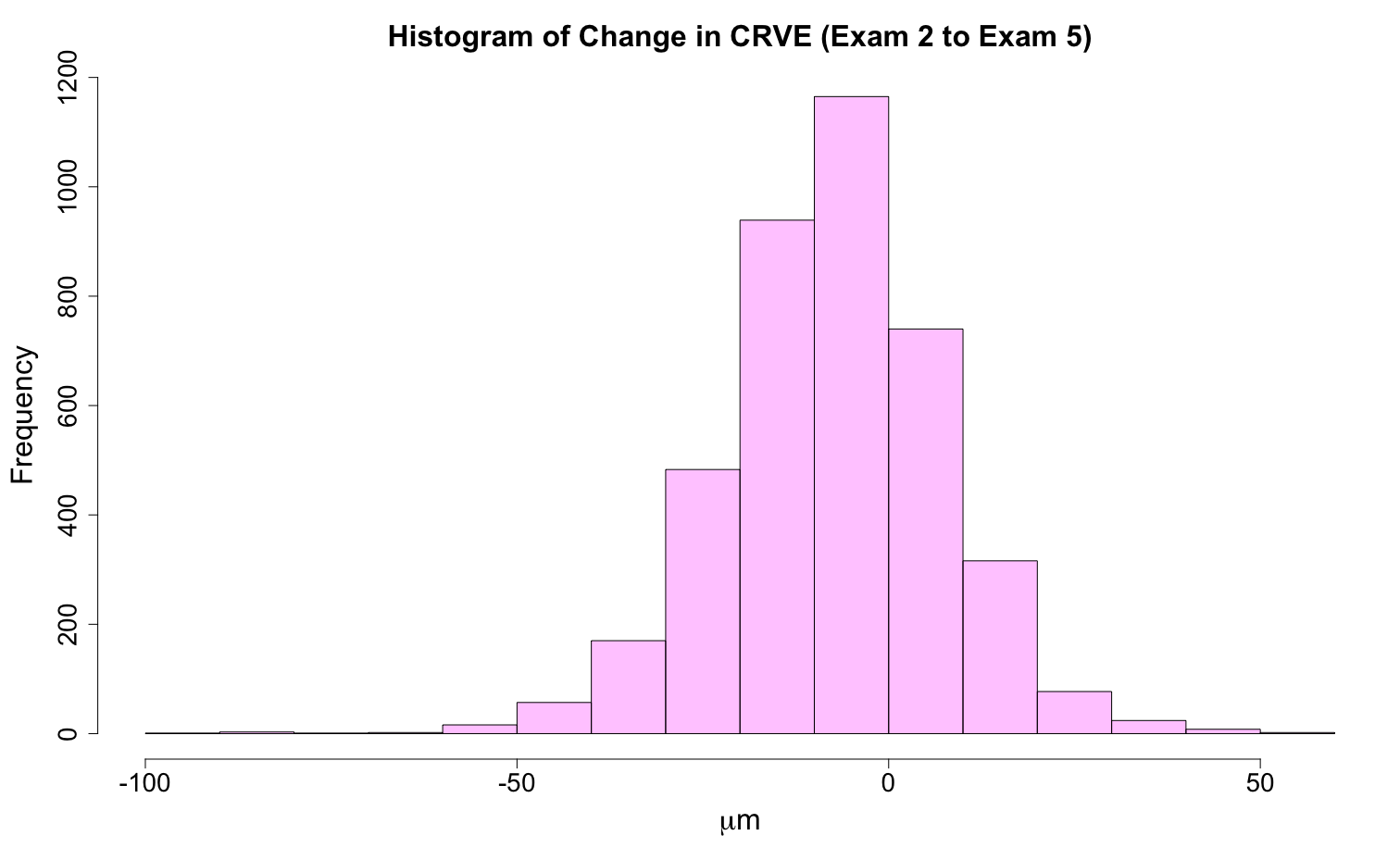


> Summary statistics for Change in CRVE (µm)

Min. 1st Qu. Median Mean 3rd Qu. Max. SD

-91.173 -16.390 -7.305 -7.559 1.658 50.720 14.8
